# Vibration of the Whole Foot Soles Surface Using an Inexpensive Portable Device to Investigate Age-Related Alterations of Postural Control

**DOI:** 10.3389/fnhum.2021.719502

**Published:** 2021-09-10

**Authors:** Lydiane Lauzier, Mohamed Abdelhafid Kadri, Emilie Bouchard, Kevin Bouchard, Sébastien Gaboury, Jean-Michel Gagnon, Marie-Pier Girard, Andréanne Larouche, Roxane Robert, Patrick Lapointe, Rubens A. da Silva, Louis-David Beaulieu

**Affiliations:** ^1^Laboratoire de Recherche Biomécanique & Neurophysiologique en Réadaptation Neuro-Musculo-Squelettique (Lab BioNR), Centre Intersectoriel en Santé Durable, Université du Québec à Chicoutimi, Chicoutimi, QC, Canada; ^2^Laboratoire d’Intelligence Ambiante pour la Reconnaissance d’Activités (LIARA), Centre Intersectoriel en Santé Durable, Université du Québec à Chicoutimi, Chicoutimi, QC, Canada; ^3^Centre Intégré de Santé et Services Sociaux du Saguenay-Lac-Saint-Jean (CIUSSS SLSJ), Hôpital de La Baie, Saguenay, QC, Canada

**Keywords:** aging, cutaneous vibration, exteroception, foam surface, postural control, proprioception running title

## Abstract

**Background:** Standing on a foam surface is used to investigate how aging affect the ability to keep balance when somatosensory inputs from feet soles become unreliable. However, since standing on foam also affects the efficacy of postural adjustments, the respective contributions of sensory and motor components are impossible to separate. This study tested the hypothesis that these components can be untangled by comparing changes of center of pressure (CoP) parameters induced by standing on a foam pad vs. a novel vibration (VIB) platform developed by our team and targeting feet soles’ mechanoreceptors.

**Methods:** Bipedal postural control of young (*n* = 20) and healthy elders (*n* = 20) was assessed while standing barefoot on a force platform through 3 randomized conditions: (1) Baseline (BL); (2) VIB; and (3) Foam. CoP Amplitude and Velocity in the antero-posterior/medio-lateral (AP/ML) directions and COP Surface were compared between conditions and groups.

**Findings:** Both VIB and Foam increased CoP parameters compared to BL, but Foam had a significantly greater impact than VIB for both groups. Young and Old participants significantly differed for all three Conditions. However, when correcting for BL levels of postural performance, VIB-related increase of COP parameters was no longer different between groups, conversely to Foam.

**Interpretation:** Although both VIB and Foam highlighted age-related differences of postural control, their combined use revealed that “motor” and “sensory” components are differently affected by aging, the latter being relatively unaltered, at least in healthy/active elders. The combined used of these methods could provide relevant knowledge to better understand and manage postural impairments in the aging population.

## Introduction

Aging results in structural and functional deteriorations of sensory receptors, as well as less effective neural processing of sensory afferents for motor and postural control (Perry, 2006; Atchison et al., 2008; Goble et al., 2009; Kwan et al., 2010; Seidler et al., 2010; Owsley, 2011). These changes play a key role in the increased risk of postural imbalance and falls in older adults (>65 years) (Vellas et al., 1997; Viseux, 2020), which have dreadful consequences both in human lives and healthcare costs^1^. Cutaneous mechanoreceptors located under the feet progressively lose their sensitivity with advancing age [i.e., reduced number of sensory receptors, decreased skin elasticity and a lower conduction time in the nervous system (Shaffer and Harrison, 2007; Kwan et al., 2010; Roberts and Allen, 2016; Viseux, 2020). Reduced sensitivity to cutaneous stimuli is in fact one of the earliest signs of peripheral neuropathy that develops with advancing age and in the presence of various diseases such as diabetes (Sacco et al., 2015). As a direct physical link with the ground, the foot sole is particularly well positioned to provide the postural control system with crucial sensory information about the ongoing state of balance (Viseux, 2020). Testing the integrity of feet mechanoreceptors and their contribution to postural control is thus highly relevant for an early detection of balance disorders and risk of falls in older peoples (Magnusson et al., 1990; Aboutorabi et al., 2018). However, most of the available evidence tested foot soles sensitivity with the participant lying down or seated, hence limiting our understanding of how foot sole receptors are actively engaged during postural tasks (Patel et al., 2011; Mildren et al., 2016).

Previous attempts to isolate the contribution of the foot sole during upright stance used various neuro-inhibitory methods targeting cutaneous receptors (e.g., cryotherapy, pharmacology or ischemia) (Magnusson et al., 1990; Meyer et al., 2004; Patel et al., 2011; Machado et al., 2017). However, these methods can be time-consuming, invasive and painful, and are therefore less suited for identifying sensory and postural control impairments in clinical settings. Instead, clinicians often ask patients to stand barefoot with their eyes closed on a compliant surface such as a foam mat (Cohen et al., 1993; Chiang and Wu, 1997; Patel et al., 2008; Khattar and Hathiram, 2012). The use of a soft surface intends to reduce the quality of skin mechanoreceptors’ input from the feet soles due to the decreased strength of the ground reaction force compared to a firm surface (Chiang and Wu, 1997). As a result, postural sway increases and discharge patterns from feet mechanoreceptors decrease because the postural control system re-weighted the sensory gains to rely more on the other available inputs (e.g., proprioceptive afferents from the ankles joint and muscles, vestibular system) (Fransson et al., 2007; Baltich et al., 2015). Therefore, the use of a foam mat helps clinicians interpreting the risk of falls and postural control impairments from a proprioceptive and exteroceptive standpoint. However, because the patient stands on a surface having particular viscoelastic properties, postural adjustments are also less efficient (Patel et al., 2011). Standing on a foam thus represents a more challenging environment for both sensory and motor aspects of postural control, and their distinctive impact on the global increase of body sway is inextricable.

In research, stimulation of the plantar surface of the foot by the use of focal vibration motors could offer an alternative solution to investigate the specific role of feet mechanoreceptors and sensory re-weighting mechanisms for postural control (Kavounoudias et al., 1998; Maurer et al., 2001; Thompson et al., 2011; Viseux et al., 2019). Our team recently developed the PortVIBplate, a simplified, portable and inexpensive tool to test the contribution of cutaneous mechanoreceptors to postural control (Lafontaine et al., 2020). The device exploits 40 vibration motors analogically. The software to control the platform, the 3D schematics and important information are made openly available to be reproduced by technological savvy persons (Lafontaine et al., 2020). Vibration (VIB) of feet soles with such a system has the advantage of reducing the quality of sensory inputs from mechanoreceptors without impacting the effectiveness of postural adjustments because the person is standing on a firm surface. The strong suprathreshold stimulation of the whole foot by the synchronous activation of VIB motors across the whole foot soles results in a constant bombardment of sensory receptors (Kavounoudias et al., 1998; Maurer et al., 2001; Thompson et al., 2011). By directly increasing their firing rate, foot soles mechanoreceptors are therefore less able to track and correct the course of the ongoing body sway. Contrasting the effects between VIB and foam could provide an unprecedented way for discriminating the contributions of sensory (vibration) vs. sensory + motor (foam) aspects of postural control allowing clinicians to better detect and manage sensory impairments and consequently reduce the risk of falls in older adults (>65 years).

The aim of this study was to compare the effects of vibrating the feet soles vs. standing on a foam mat on postural control in young adults and older adults (>65 years). Based on the available evidence, we hypothesized that (i) old adults will generally present higher postural imbalances compared to young adults; (ii) both the foam mat and feet vibration will increase postural oscillations compared to control condition (i.e., standing on a firm surface), but the impact will be greater for the foam because it challenges both sensory and motor aspects of postural control.

## Materials and Methods

### Participants

Forty male and women aged from 22 to 87 years old were recruited to participate in the experiment. They were divided into 2 groups, young (*n* = 20) and old (*n* = 20) based on their age (i.e., young group: 18–30 years; old group: >65 years). Age, morphological characteristics and details about the level of their physical activity are presented in Table 1. Based on the inclusion criteria, the recruited participants were in good general health, community-dwelling and able to walk independently without a walking-aid. Exclusion criteria included any functional impairment related to cognitive, neurological, musculoskeletal, vestibular disorders, cardiovascular diseases and ankle, knee and hip injuries in the past 2 years. Research ethical approval was obtained prior to recruitment and participants gave their written informed consent in accordance with the Declaration of Helsinki and the local Ethics Committee.

**TABLE 1:** Participant’s characteristics.

	Young *N* = 20	Old *N* = 20	Significant difference between groups
**Characteristics**			
**Age (years):**			
Mean ± SD	24.85 ± 2.37	73.8 ± 5.72	*p* = 0.00
Gender (*n* = women/male)	9/11	9/11	*p* = 0.62
**Height (cm):**			
Mean ± SD	171.41 ± 9.43	166.68 ± 9.1	*p* = 0.11
**Weight (lbs):**			
Mean ± SD	162.5 ± 35.33	157.35 ± 29.39	*p* = 0.61
**Physical activity level (min/week):**			
Mean ± SD	374.2 ± 297.08	147.4 ± 65.81	*p* = 0.00

### Experimental Procedure

Anthropometric measures were taken during the first session and the Global Physical Activity (GPAQ_v2_) questionnaire (Bull et al., 2009) was self-administered to collect information regarding the level of physical activity at work and in leisure time. The experimental session (60 min) assessed postural control in bipedal stance in three randomized conditions (10 s/condition; 5 trials/condition): (i) Baseline (standing on the vibration platform but without stimulation), (ii) VIB (cutaneous vibration applied to the whole surface of both feet), (iii) Foam (standing on a 20″ × 16″ × 2.5″ balance pad, Airex Compagny, Switzerland). Duration of the postural measures in all conditions is established according to the duration of vibratory stimulation which, in a similar context (i.e., disruption of muscle spindles by tenon vibration), presents a good reliability for the CoP-based postural parameters (Kadri et al., 2020). Vibration was delivered at 50 Hz by the 40 vibrators (20/foot) of the portable vibration platform (*PortVIBplate*—see Lafontaine et al. (2020)). For all conditions, participants were asked to stand barefoot with eyes blindfolded and the arms along the body, and the feet position was standardized using marks on the *PortVIBplate* and Foam mat. The *PortVIBplate* and the foam mat were placed on a force platform (BIOMEC400, EMG System do Brazil, Ltda., SP) [for the characteristics see Kadri et al. (2020)], to measure variables of postural control based on linear parameters of the center of pressure (CoP). Brief rest period of 1 min between each trial and each condition allowed participants to rest and remove the blindfold. An investigator stood close to the participant to ensure its security during the tests. To control for potentially disruptive sounds and other distractions, the experimenters and participants were instructed to avoid conversations and make abrupt movements.

### Data Analysis

The vertical ground reaction force data from the force platform was sampled at 100 Hz. All force signals were filtered with a Butterworth second order low-pass 35 Hz filter (Kadri et al., 2020). Data was converted into stabilometric analyses using the BIOMEC software combined with MATLAB routines (The Mathworks, Natick, MA, United States). The Amplitude (the absolute distance between the max and min center of pressure displacement, in cm), Velocity (sum of the cumulated CoP displacement divided by the total time, in cm s^–1^) in the anteroposterior (AP) and mediolateral (ML) directions of movement and Surface (90% confidence ellipse, in cm^2^) were calculated from CoP data.

### Statistical Analysis

Data normality and the absence of outliers were confirmed with the Shapiro–Wilk test and visual screening of box-and-whisker plots. Participant’s characteristics were compared with the Unpaired *T*-Test (age, height, weight, and physical activity level) and the Chi-square test (gender). A repeated measures ANOVA model was applied with factors *Group* (young; old) and *Conditions* (BL, VIB, and Foam). In cases of a significant interaction between factors, pair-wise Bonferroni tests adjusted for multiple comparisons were realized. Statistical analysis was done using the SPSS version 20 program (Armonk, NY, United States) with a significant alpha risk below 0.05.

## Results

The young and old groups did not differ in terms of gender, height and weight, but were significantly different in their age and level of physical activity (young participants being more active than older—cf. Table 1).

### Effects of Groups and Experimental Conditions

The analysis of variance found significant interactions between factors *Groups* and *Conditions* for all CoP parameters: AP Amplitude [*F*_(2;76)_ = 7.68; *P* = 0.01]; ML Amplitude [*F*_(2;76)_ = 18.65; *P* < 0.000]; AP Velocity [*F*_(2;76)_ = 4.91; *P* = 0.01]; ML Velocity [*F*_(2;76)_ = 22.22; *P* < 0.000]; CoP Surface [*F*_(2;76)_ = 21.43; *P* < 0.000]. As shown in Table 2, *post hoc* comparisons found that all CoP parameters were significantly higher (*p* values < 0.01) for old compared to young participants across the three experimental conditions (BL, VIB, and Foam). Also, both groups showed significantly higher CoP parameters (*P* values < 0.01) with mostly large (>0.80) effect sizes (Cohen’s D, G^∗^Power 3.1 Software) for Foam vs. BL, VIB vs. BL and Foam vs. VIB (Table 3).

**TABLE 2:** Postural parameters expressed in mean ± standard deviation for each group and experimental conditions.

	Groups					
	Young			Old		
	Conditions					
Postural parameters	Baseline	VIB	Foam	Baseline	VIB	Foam
Amplitude AP (cm)	1.20 ± 0.33	1.41 ± 0.39^a^	2.79 ± 0.64^a,b^	1.40 ± 0.43	1.72 ± 0.54^a^	3.76 ± 1.16^a,b^
Amplitude ML (cm)	1.49 ± 0.43	2.10 ± 0.62^a^	3.41 ± 0.63^a,b^	1.88 ± 0.47	2.46 ± 0.74^a^	5.03 ± 1.01^a,b^
Velocity AP (cm/s)	1.07 ± 0.17	1.20 ± 0.18^a^	1.85 ± 0.49^a,b^	1.28 ± 0.33	1.38 ± 0.29^a^	2.43 ± 0.73^a,b^
Velocity ML (cm/s)	1.02 ± 0.32	1.55 ± 0.43^a^	2.19 ± 0.53^a,b^	1.38 ± 0.44	1.99 ± 0.69^a^	3.77 ± 1.06^a,b^
Surface (cm^2^)	1.66 ± 0.84	2.63 ± 1.63^a^	8.70 ± 3.53^a,b^	2.42 ± 1.28	3.72 ± 2.05^a^	16.75 ± 6.72^a,b^

*All conditions are statistically different between Young and Old groups (p < 0.01). ^a^denotes significant difference with Baseline (p < 0.01). ^b^denotes significant difference between Foam and VIB conditions (p < 0.01). VIB, Cutaneous vibration with the PortVIBplate.*

**TABLE 3:** Effect sizes (Cohen’s D) between different conditions for each group in all postural parameters.

	Baseline vs VIB		Baseline vs Foam		VIB vs Foam	
	
	Young group	Old group	Young group	Old group	Young group	Old group
**Postural parameters**						
Amplitude AP (cm)	0.57	0.64	2.86	2.32	2.47	2.02
Amplitude ML (cm)	1.10	0.89	3.44	3.59	2.09	2.83
Velocity AP (cm/s)	0.74	0.32	1.81	1.81	1.51	1.64
Velocity ML (cm/s)	1.36	1.00	2.53	2.59	1.31	1.91
Surface (cm2)	0.68	0.72	1.36	2.31	1.23	2.18

Since Young and Old groups differed at BL, a supplementary analysis was realized to compare the impact of VIB and Foam between groups when correcting for BL levels of CoP parameters. Specifically, CoP parameters obtained during VIB and Foam conditions were expressed as ratios of BL level (i.e., VIB BL; Foam BL) and Young and Old groups were compared using Unpaired *T*-Tests. The analysis revealed that the following Foam/BL ratios were significantly higher for Old vs. Young participants: ML Velocity (*p* = 0.002); CoP Surface (*p* = 0.014). No significant differences between groups were found for VIB/BL ratios.

## Discussion

The present study compared the postural responses resulting from two different techniques aiming at altering the sensitivity of plantar mechanoreceptors in healthy young and old participants. Our results confirmed that significant but distinctive effects were found between these two techniques, suggesting different underlying mechanisms of sensorimotor integration and postural control. Using both vibratory and foam approaches in clinical practice could provide rich and complementary knowledge for better managing postural impairments and risk of falls in the older adults (>65 years).

We are not the first to use plantar cutaneous vibration to investigate mechanisms of postural control. However, previous studies vibrated restricted zones of the foot, such as the forefoot or the heels (Kavounoudias et al., 1998, 1999, 2001; Roll et al., 2002). Vibrating the forefoot soles mimics a forward change in the CoP position under the feet. This false proprioceptive signal is interpreted as a fall, and a rapid backward postural reaction occurs to maintain balance. In 1999, Kavounoudias et al. (1999) did test the impact of co-vibrating the two heels and two forefoot zones in nine young adults. They reported a slight but non-significant increase of COP parameters compared to baseline value without VIB. Conversely, our study found significant increases of all COP parameters when applying VIB, even in the youngest participants. Discrepancies between our respective results could be ascribed to the different methods used and sample size, especially VIB duration which was longer in the present work [10 vs. 2.5 s in Kavounoudias et al. (1999)]. Also, they employed 4 VIB motors covering restricted parts of the foot (heels and metatarsal heads), whereas our 40 vibrators essentially covered the whole foot soles, even the toes which have the highest density of cutaneous mechanoreceptors (Viseux et al., 2019). In addition, to the best of our knowledge no study used VIB of the whole foot to compare postural effects between young and older populations. A few studies did use other vibration techniques, such as tendinous vibration of ankle muscles (Hay et al., 1996; Teasdale and Simoneau, 2001; Abrahamova et al., 2009). It was found that these proprioceptive-to-motor postural reactions were greater in healthy older vs. younger adults, suggesting that age does not significantly impact the interpretation of proprioceptive feedback, but instead limits the ability to adapt to the disruptive sensory signal and reach a new “stabilized” posture during VIB stimulation (Abrahamova et al., 2009).

Conversely, we rather used vibration as a “directionless” increase in mechanoreception firing of the whole sole surface, and found that the determinants of postural stability (i.e., the Amplitude of CoP), postural control (the Velocity of CoP) and postural performance (i.e., the Surface of CoP) (Paillard and Noe, 2015) were markedly increased for both groups. This finding confirms that the spatial and temporal information about the pressure variations exerted under the feet were significantly altered by vibration (Kavounoudias et al., 1998, 1999), highlighting the privileged position of the feet soles as the only physical link between the body and the ground (Viseux, 2020). Plantar skin mechanoreceptors play a key role in regulating mechanisms of postural control by transmitting crucial information about the body’s relation with the environment (Patel et al., 2008; Viseux, 2020). The fact that mechanical vibration increased postural oscillations for both young and older adults support the relevance and effectiveness of this approach to study sensory processing and re-weighting mechanisms. Sensory re-weighting can be observed in the presence of an altered sensory signal (like it is the case when vibrating skin mechanoreceptors) and consists of dynamic inhibitory/excitatory mechanisms resulting in a decreased gain from the altered signal and increased reliance on the other unaltered sensory systems (Mahboobin et al., 2009).

As expected, our results showed a greater increase in all CoP postural parameters while standing on a foam surface in both young and old people (Table 2). These results are consistent with previous studies having found a decreased performance of postural control when standing with eyes closed on a foam surface vs. on a solid surface (Cohen et al., 1993; Chiang and Wu, 1997; Patel et al., 2008, 2011). Because our results found a significantly higher impact of foam vs. feet vibration for both groups (Table 2), it can be hypothesized that the relative contribution of “motor” disturbances caused by standing on an unsteady environment adds a significant impact to the reduced reliability of somatosensory information from the feet. Instead of VIB, Patel et al. (2011) applied a cooling technique targeting fast-adapting mechanoreceptors of foot soles and also concluded that foam-induced effect on postural control involves different mechanisms that remains to be elucidated. Therefore, we highly recommend that observations from the foam condition consider both the sensory and motor disturbances as a whole, because only cutaneous-specific methods such as VIB offer a restricted mean of altering mechanoreceptors’ function. However, by being more challenging the foam mat might serves as a better screening tool for ruling out postural control impairments than the vibratory platform. Overall, the use of both approaches (Foam and VIB with the *PortVIBplate*) in clinical practice could provide a more in-depth evaluation of sensory vs. motor impairments in an individual presenting with postural imbalance.

Another interesting finding concerns group comparisons between young and older participants. During baseline condition, postural parameters were significantly higher in the old group compared to the young group, which is in line with the literature having described a progressive deterioration of postural control with age (Lord et al., 2018). Aging has been shown to cause a decreased sensitivity of somatosensory receptors, lower conduction times within the nervous system, altered efficacy of sensorimotor processing mechanisms and delayed/weaker postural adjustments (Shaffer and Harrison, 2007; Viseux, 2020). In particular, Pacini’s corpuscles exhibit a reduction in their number and a decrease in their sensitivity to cutaneous vibration and other mechanical stimuli (Shaffer and Harrison, 2007). These age-related changes could in part explain the different performances at baseline between young and old groups. However, the significant increase in postural sway caused by vibration underlines that cutaneous afferents from the feet are still contributing significantly to maintain balance, at least in healthy and physically active older adults (>65 years) (Vellas et al., 1997; Kristinsdottir et al., 2001). Interestingly, when correcting for BL differences with the use of Foam/BL and VIB/BL ratios, we found that only Foam resulted in significantly higher postural disturbances for Old vs. Young participants. Because VIB had no such age-related contrast, it can be argued that mechanisms involved in re-weighting of sensory gains toward more reliable inputs (e.g., proprioceptive afferents from the ankles joint and muscles, vestibular systems) (Fransson et al., 2007; Baltich et al., 2015) are relatively unaffected in healthy elders. Instead, postural impairments observed when sanding on the foam pad are probably resulting to a greater extend to less effective motor adjustments (ex: appropriate strength and timing of leg muscle contractions).

As mentioned above, this new knowledge brought by our work enables an improved analysis of the specific contribution of somatosensory afferents to postural control. This is highly relevant for both research and clinical purposes, for instance to foster our understanding of sensory reweighting mechanisms and how there are affected by peripheral neuropathy and other age-related changes within the nervous system. For example, if no change of postural sway is observed when vibrating the feet soles it can be hypothesized that mechanoreceptors are severely impaired. Then, depending on the performances at baseline (and also with the foam mat), it can be concluded that the postural control system was able to rely on other sensory systems, or not. Future studies could also investigate whether postural oscillations are progressively re-stabilizing when applying a prolonged vibration, which would signify that the postural control network can undergo plastic adaptation and motor learning in the presence of a challenging / unreliable sensory environment. Future studies should investigate similar mechanisms but in persons presenting with balance and sensorimotor disorders, and in other postural tasks such as the semi-tandem or unipedal stance. Interestingly, recent publications highlighted the potential clinical interest of using skin vibration as a novel neurostimulation therapy for sensory and postural control retraining (Zhou et al., 2016; Viseux et al., 2019). Our vibration system could therefore serve both to identify individuals with altered foot sole / sensory-reweighting functions and provide therapeutic options to reduce the risk of fall in elders and other populations suffering from peripheral neuropathy.

Our results are mainly limited by the use of an inexpensive vibration platform, the PortVIBplate designed to be clinical-friendly (Lafontaine et al., 2020). VIB frequency in the present study was set at 50 Hz according to the technical charts provided by the manufacturer’s motors. Since the rotational movement of motors can be altered by several factors (e.g., technical quality of the motor, compliance of the surface in contact with the motor, variation in the electrical current which is separated into 40 motors, amount/modulation of pressure exerted by the foot), the actual frequency transmitted to the subject’s skin during weight-bearing was impossible to verify. During pilot tests realized when developing the PortVIBplate, we found random variations in the observed frequency which sometimes peaked at ± 20 Hz around the targeted 50 Hz. Even though we were able to find significant effects with our VIB parameters, it is impossible to know whether different findings could have been obtained if using different parameters (i.e., frequency or duration), knowing for instance that higher frequencies tend to induce greater postural effects (Kavounoudias et al., 1999, 2001). The impact of better controlling and modulating VIB parameters should be investigated further in the future. There is a clear need for developing closed-loop control systems able to measure and possibly apply online corrections of VIB parameters to ensure the methodological robustness of VIB research. Also, we recruited healthy and physically active older adults (>65 years) that are not representative of the general population, hence potentially underestimating the impact of age and comorbidities on the postural control system. Finally, guidelines about CoP measurements recommend at least 10 s for unipedal balance (Lin et al., 2008), and 10–20 s and more for the bipodal stance like in our study for reaching high reliability indices of CoP data (Parreira et al., 2013), but we preferred to restrict vibration to 10 s to replicate published protocols in the field of VIB-induced postural effects (Kadri et al., 2020). Future works should consider trying different vibration durations in order to determine the protocol offering the best balance between reliability, time-consumption and fatigue build-up.

In conclusion, our study provided the first evidence of a contrasting effect between two postural tests of sensory-reweighting (Foam, cutaneous VIB). While both approaches induced significant postural imbalances and were able to highlight age-related differences, we argue that only VIB of the whole plantar surface can specifically alter the sensory feedback from plantar soles mechanoreceptor. Using this specificity of VIB, we found that “motor” rather than “sensory” components involved postural control was primarily affected by aging. The foam mat might better serve as a screening tool, as the postural imbalances likely encompass both sensory and motor disturbances related to standing on an unsteady environment. Using the two methods in clinical practice appear highly complementary, for instance to investigate the integrity of proprioception coding in the presence of diabetic peripheral neuropathy (VIB), or to evaluate the risk of fall in unsteady environment such as snowy/sandy grounds (Foam).

## Data Availability Statement

The raw data supporting the conclusions of this article will be made available by the authors, without undue reservation.

## Ethics Statement

The studies involving human participants were reviewed and approved by Comité d’Éthique à la Recherche Avec des Êtres Humains—Université du Québec à Chicoutimi. The patients/participants provided their written informed consent to participate in this study.

## Author Contributions

LL, J-MG, M-PG, AL, and RR contributed to the conception and design of the study, realized the experiments, and statistical analysis. LL wrote the first draft of the manuscript. MK and EB helped in the experiments, statistical analysis, and redaction of the manuscript. PL, KB, and SG developed the PortVIBplate technology tested in the study and contributed to the redaction. RS and L-DB trained and supervised the students involved throughout the study. As senior author and PI of this project, L-DB oversaw all aspects of the study. All authors contributed to manuscript revision, read, and approved the submitted version.

## Conflict of Interest

The authors declare that the research was conducted in the absence of any commercial or financial relationships that could be construed as a potential conflict of interest.

## Publisher’s Note

All claims expressed in this article are solely those of the authors and do not necessarily represent those of their affiliated organizations, or those of the publisher, the editors and the reviewers. Any product that may be evaluated in this article, or claim that may be made by its manufacturer, is not guaranteed or endorsed by the publisher.

## Footnotes

1
www.who.int/mediacentre/factsheets/fs344/fr/
